# Neutering Practices, Reproductive Management, and Clinical Findings in European Endurance Sled Dogs: A Field Study from the Lekkarod^®^ Event

**DOI:** 10.3390/ani16213457

**Published:** 2026-11-02

**Authors:** Vinciane Roger, Dominique Grandjean, Milagros Benito

**Affiliations:** 1 École Nationale Vétérinaire d’Alfort (EnvA), Université Paris-Est, 94700 Maisons-Alfort, France; 2 Institut NOSAÏS, École Nationale Vétérinaire d’Alfort (EnvA), Université Paris-Est, 94700 Maisons-Alfort, France; 3 Department of Animal Medicine and Surgery, CEU Faculty of Veterinary Medicine, Universidad CEU Cardenal Herrera, 46115 Valencia, Spain

**Keywords:** sled dogs, gonadectomy, neutering, reproductive management, canine athletes, endurance racing, veterinary sports medicine, musculoskeletal health, metabolic abnormalities, working dogs

## Abstract

Decisions regarding neutering in athletic sled dogs involve a complex balance between health, behaviour, performance, breeding objectives, and management considerations. However, evidence regarding neutering practices and their relationship with health outcomes in actively competing sled dogs remains limited. To identify factors associated with neutering status and musher-reported motivations for neutering or maintaining dogs intact, and to evaluate the occurrence and distribution of clinical findings according to reproductive status in sled dogs participating in the 2023 Lekkarod^®^ event. A total of 599 sled dogs registered for the 2023 Lekkarod^®^ event, including participants in the main endurance race and the associated Kraft tournament, were included. Information regarding age, sex, breed, neutering status, and owner-reported motivations was collected using a structured questionnaire. Multivariable logistic regression was used to identify factors associated with neutering status. Clinical findings recorded during veterinary examinations conducted throughout the main Lekkarod race were classified and analysed according to reproductive status. Overall, 28.0% (168/599) of dogs were neutered. Neutering was significantly associated with female sex, older age, and breed category, whereas team position was not associated with reproductive status. The most frequently reported reasons for neutering were reproductive management, behavioural management, and disease prevention, whereas breeding purposes, preservation of genetic lines, performance-related considerations, and ethical concerns were the principal motivations for maintaining dogs intact. A total of 82 clinical findings were recorded, corresponding to 13.7% of the study population. Reproductive status was not associated with the overall occurrence of clinical abnormalities. However, the distribution of clinical finding categories differed significantly according to reproductive status (χ^2^ = 7.564, *p* = 0.023). Intact dogs presented proportionally more metabolic/systemic abnormalities, whereas neutered dogs presented proportionally more musculoskeletal findings. The observed distribution of musculoskeletal findings was not explained by age alone. In this population of actively competing European sled dogs, neutering was relatively uncommon and reflected a combination of reproductive management, health, breeding, and performance-related considerations. Although reproductive status was not associated with the overall occurrence of clinical abnormalities during competition, it was associated with differing patterns of clinical findings. Intact dogs presented proportionally more metabolic abnormalities, whereas neutered dogs presented proportionally more musculoskeletal findings. These findings support an individualised approach to reproductive management in canine athletes and warrant further prospective investigation of the relationships among reproductive status, metabolic adaptation, musculoskeletal health, and athletic performance.

## Simple Summary

Endurance sled dogs are highly trained canine athletes exposed to substantial physical and environmental demands during competition. Decisions regarding neutering in these dogs are influenced not only by health and reproductive management considerations, but also by breeding objectives, kennel management, and perceptions regarding athletic performance. Despite the practical importance of these decisions, little information is available regarding neutering practices in actively competing sled dogs. This study evaluated 599 sled dogs participating in the 2023 Lekkarod^®^ event in France. Information regarding age, sex, breed, neutering status, and musher-reported motivations for neutering or maintaining dogs intact was collected. Veterinary examinations performed during the main race were also used to investigate the occurrence of clinical abnormalities. Overall, 28% of dogs were neutered, with neutering being more common in females and older dogs. The most frequently reported reasons for neutering were reproductive management and disease prevention, whereas breeding objectives, preservation of genetic lines, performance-related considerations, and ethical concerns were common reasons for maintaining dogs intact. Approximately 14% of dogs presented at least one clinical abnormality during competition. Although neutering status was not associated with the overall occurrence of clinical abnormalities, the pattern of findings differed according to reproductive status. Intact dogs presented proportionally more metabolic abnormalities, whereas neutered dogs presented proportionally more musculoskeletal findings. These results support an individualised approach to reproductive management in canine athletes and provide new information regarding health monitoring in endurance sled dogs.

## 1. Introduction

Sled dogs participating in long-distance and multistage races represent a unique population of canine endurance athletes. These dogs are exposed to prolonged physical exercise, substantial training loads, challenging environmental conditions, and intensive management practices aimed at maintaining both health and athletic performance. Physiological studies have demonstrated marked adaptations in endurance sled dogs compared with companion dogs, particularly in energy metabolism, exercise tolerance, and recovery from prolonged exertion [1,2,3]. The demanding nature of sled dog racing, including environmental stressors, prolonged exercise, nutritional challenges, and cumulative fatigue, has been comprehensively reviewed and remains an important welfare and sports medicine concern [4].

Endurance racing may challenge multiple physiological systems. Previous investigations have identified aspiration pneumonia, gastric ulceration, gastrointestinal disease, and exercise-associated muscle disorders among the main causes of morbidity and mortality in racing sled dogs [5]. Endoscopic studies have also demonstrated a high prevalence of gastric lesions in apparently healthy racing dogs following competition [6]. In addition, orthopaedic injuries represent one of the most common reasons for race withdrawal, particularly involving the shoulder and carpal regions [7]. More recently, metabolomic studies have revealed profound exercise-induced alterations in substrate utilisation, amino acid metabolism, and energy regulation, highlighting the extraordinary physiological adaptations required for endurance racing [8]. Together, these findings emphasise the importance of monitoring factors that may influence health outcomes in canine endurance athletes.

Among the many management decisions faced by mushers and kennel owners, reproductive management is particularly important. Neutering, including ovariohysterectomy in females and orchiectomy in males, is widely practised in companion dogs for population control, reproductive management, and prevention of selected reproductive disorders [9,10]. However, decisions regarding gonadectomy in working and sporting dogs often involve additional considerations related to athletic performance, breeding value, behaviour, welfare, and long-term health [11,12].

Growing attention has been directed towards the potential long-term consequences of gonadectomy. Gonadal hormones play important roles in skeletal maturation, musculoskeletal development, metabolism, and behaviour [13]. Several studies have reported associations between neutering and increased risks of orthopaedic disorders, including cranial cruciate ligament disease, hip dysplasia, and elbow dysplasia in predisposed breeds [14,15,16]. Associations have also been described with altered body condition, obesity, selected neoplastic diseases, and behavioural disorders, although the magnitude of these effects appears to vary among breeds and study populations [13,17,18]. In a large retrospective cohort, gonadectomy was associated with an increased risk of overweight and obesity [19], while a recent systematic review and meta-analysis reported significantly increased odds of cranial cruciate ligament disease among gonadectomised dogs [20].

Importantly, most available evidence originates from companion animal populations. Whether these findings can be extrapolated to highly conditioned canine athletes remains uncertain. Working and sporting dogs are exposed to unique physiological demands and management practices that may modify both the benefits and risks associated with gonadectomy. Furthermore, owners and trainers of working dogs frequently express concerns regarding potential effects of neutering on motivation, trainability, endurance, and willingness to work [12,21]. These concerns are supported by owner-based studies indicating that dogs intended for working activities are less likely to be neutered than companion animals [11]. Such considerations may be particularly relevant in sled dog racing, where athletic performance, breeding objectives, and long-term health frequently coexist within the same decision-making process.

Reproductive decisions within sled dog kennels are further complicated by practical and strategic considerations. Many kennels maintain multiple intact males and females, requiring management of oestrus cycles and prevention of unintended breeding while preserving social stability and breeding opportunities. At the same time, selective breeding programmes are fundamental to maintaining desirable performance traits, behavioural characteristics, and genetic diversity within successful kennels [22,23]. Consequently, reproductive management decisions often reflect a complex interaction of health, behavioural, athletic, genetic, ethical, and management-related factors.

Although discussion regarding the welfare implications of neutering has intensified in recent years [9,13], empirical evidence derived directly from actively competing sled dogs remains scarce. Little information is available regarding neutering practices, the motivations underlying reproductive management decisions, or the possible relationship between reproductive status and clinical findings recorded under real racing conditions. This knowledge gap is particularly relevant given the unique athletic and management demands experienced by sled dog populations.

The 2023 Lekkarod^®^ event, including both the main endurance race and the associated Kraft tournament, provided a valuable opportunity to investigate these issues in a large population of actively competing European sled dogs. Therefore, the aims of the present study were to (1) identify factors associated with neutering status, (2) describe musher-reported motivations for neutering or maintaining dogs intact, and (3) evaluate the occurrence and distribution of clinical findings during competition according to reproductive status.

## 2. Materials and Methods

### 2.1. Study Design and Population

This observational cross-sectional study investigated factors associated with neutering status, reproductive management decisions, and the occurrence of clinical findings in sled dogs participating in the 2023 Lekkarod^®^ event in France.

The study population consisted of 599 client-owned sled dogs, including 270 dogs participating in the main Lekkarod endurance race and 329 dogs participating in the associated Kraft tournament. All dogs were officially registered for competition and were considered fit to participate according to race regulations.

Data were obtained from official race registration records and veterinary information collected during routine health assessments performed as part of race organisation. For analyses involving reproductive status, only dogs with complete information regarding sex and neutering status were included.

### 2.2. Data Collection and Variables

For each dog, the following information was recorded: age at the time of the event, sex, breed category, competition category, neutering status and clinical findings.

Dogs with missing or uncertain information regarding reproductive status were excluded from analyses involving neutering.

Additional information was available for dogs participating in the 2023 Lekkarod^®^ event, including team position within the sled formation, participation group (main Lekkarod race or Kraft tournament), and findings from veterinary examinations performed before and during competition.

### 2.3. Reproductive Management Decisions

As part of the mandatory pre-race veterinary examination, mushers were asked to identify the principal reason underlying the decision to neuter a dog or maintain it intact.

Reported motivations were subsequently classified using a predefined coding framework reflecting the most common themes encountered in sled dog management. Categories included reproductive management, disease prevention, behavioural management, performance-related considerations, breeding strategy, ethical considerations, and medical indications.

Only the primary motivation reported for each dog was retained for analysis. Frequencies of motivation categories were calculated and subsequently grouped into broader categories for descriptive presentation.

### 2.4. Team Position and Race Participation

Mushers reported the position most frequently occupied by each dog within the team. Positions were classified as lead, swing/team, or wheel.

Because lead and wheel dogs fulfil distinct functional roles within the team, additional dichotomous variables indicating lead position (yes/no) and wheel position (yes/no) were generated for subsequent analyses.

Race records were also used to identify the competition group in which each dog participated.

### 2.5. Veterinary Examinations and Classification of Clinical Findings

All dogs participating in the competition underwent routine veterinary examinations before and during the race as part of standard competition procedures and welfare monitoring.

Clinical findings identified during these assessments were recorded in a dedicated database containing information on dog identity, age, sex, breed, neutering status, stage of the race at the time of examination, clinical diagnosis, and treatment when applicable.

Individual abnormalities were initially recorded according to their clinical diagnosis. For the purposes of the present study, clinical findings were subsequently grouped into three categories:

Traumatic injuries, including wounds, harness-related lesions, and paw-pad abrasions;

Musculoskeletal disorders, including lumbar, triceps, psoas, and shoulder abnormalities;

Metabolic/systemic conditions, including fatigue, diarrhoea, and cardiac abnormalities.

For descriptive analyses, each recorded clinical finding was considered individually. For inferential analyses evaluating the occurrence of abnormalities during competition, dogs were classified dichotomously according to the presence or absence of at least one recorded clinical abnormality.

### 2.6. Statistical Analysis

Descriptive statistics were used to summarise the characteristics of the study population. Continuous variables are presented as mean ± standard deviation (SD) and range, whereas categorical variables are presented as frequencies and percentages.

Age was additionally categorised into three groups (young, middle-aged, and old) according to tertiles of the age distribution.

Factors associated with neutering status were evaluated using multivariable binary logistic regression. Neutering status (neutered versus intact) was included as the dependent variable. Candidate explanatory variables included participation group (main Lekkarod race or Kraft tournament), competition category, sex, breed category, lead position, wheel position, and age group. Model fit was assessed using the Hosmer–Lemeshow goodness-of-fit test, and explained variation was estimated using the Nagelkerke R^2^ coefficient. Results are reported as odds ratios (OR) and associated *p*-values.

For the analysis of clinical findings recorded during the main Lekkarod race, frequencies and percentages were calculated for each clinical finding category. Associations between neutering status and the occurrence of at least one clinical abnormality were evaluated using Pearson’s chi-square test and Fisher’s exact test when appropriate.

To investigate whether the distribution of clinical finding categories differed according to reproductive status, traumatic, musculoskeletal, and metabolic/systemic findings were compared between neutered and intact dogs using Pearson’s chi-square test.

An additional exploratory chi-square analysis was performed to evaluate whether age group could explain the distribution of musculoskeletal findings according to reproductive status.

All statistical analyses were performed using IBM SPSS Statistics (version 29; IBM Corp., Armonk, NY, USA). Statistical significance was established at *p* < 0.05.

### 2.7. Ethical Considerations

All information used in this study was collected as part of routine race registration procedures and standard veterinary care performed during competition. No experimental interventions or procedures were conducted specifically for research purposes.

Owners or authorised handlers provided consent for the use of anonymised data for scientific purposes. The study was considered exempt from formal ethical review because all examinations and clinical assessments formed part of routine veterinary practice and welfare monitoring during competition and did not involve procedures beyond standard clinical care.

## 3. Results

### 3.1. Population Characteristics

A total of 599 sled dogs registered for the 2023 Lekkarod^®^ event were included in the study. The population comprised dogs participating in both the main Lekkarod endurance race (*n* = 270, 45.1%) and the associated Kraft tournament (*n* = 329, 54.9%). Overall, 325 dogs (54.3%) were male and 274 (45.7%) were female. The mean age was 1782 ± 841 days (4.9 ± 2.3 years), ranging from 555 to 4284 days. A total of 168 dogs (28.0%) were neutered, whereas 431 (72.0%) remained reproductively intact. Competition categories were distributed between NB (319/599, 53.3%) and OP (280/599, 46.7%).

The study population was predominantly composed of Siberian Huskies (312/599, 52.1%), Alaskan Huskies (166/599, 27.7%), and European Sled Dogs (100/599, 16.7%), which together accounted for 96.5% of all enrolled dogs. The remaining 21 dogs (3.5%) included Groenendaels, Samoyeds, Dobermans, Pointer-type dogs, and mixed breeds.

The demographic and competition characteristics of the study population are summarised in Table 1.

### 3.2. Team Position Within the Sled

Information regarding team position was available for 550 dogs (91.8%). Among these, 196 dogs (35.6%) were classified as swing/team dogs, 192 (34.9%) as wheel dogs, and 162 (29.5%) as lead dogs.

Lead dogs are responsible for navigation and responsiveness to commands, whereas wheel dogs provide pulling power and stability immediately in front of the sled. Swing/team dogs occupy intermediate positions and contribute primarily to traction and team cohesion. Given the distinct functional demands associated with lead and wheel positions, dichotomous variables indicating lead position (yes/no) and wheel position (yes/no) were generated for subsequent analyses.

The distribution of team positions is summarised in Table 2.

### 3.3. Reasons for Neutering and Maintaining Dogs Intact

Information regarding reproductive management decisions was available for 592 dogs. Reported motivations differed according to reproductive status.

Among dogs maintained intact, the most frequently reported motivations were breeding and genetic preservation, no perceived need for intervention, performance-related considerations, and lack of perceived benefit associated with neutering. Additional motivations included ethical concerns regarding elective gonadectomy and other miscellaneous reasons.

Among neutered dogs, the most frequently reported motivations were oestrus and reproductive management, behavioural management, disease prevention, group-dynamics management, and medical indications.

For improved readability, motivations for maintaining dogs intact and motivations for neutering are presented separately in Table 3 and Table 4.

### 3.4. Relationship Between Sex and Team Position, and Neutering Status

Sex distribution differed significantly according to team position. Lead dogs were proportionally more often female, whereas wheel positions were predominantly occupied by male dogs. A strong association was identified between sex and team position (Pearson’s χ^2^ = 123.16, df = 1, *p* < 0.001), corresponding to a large effect size (φ = 0.47). Similar relationships remained significant when analyses were stratified by sex (both *p* < 0.001).

In contrast, reproductive status was not significantly associated with either lead position (Pearson’s χ^2^ = 0.164, df = 1, *p* = 0.686) or wheel position (Pearson’s χ^2^ = 1.173, df = 1, *p* = 0.279). Therefore, no evidence was found that neutering status was related to the functional role occupied by dogs within the team.

Overall, sex was strongly associated with the distribution of working positions within the sled team, whereas neutering status was not. These results suggest that assignment of dogs to specific functional roles is primarily influenced by sex-related characteristics and working function rather than reproductive status.

### 3.5. Pathologies Recorded During Lekkarod 2023

A total of 82 clinical findings were recorded during the competition, corresponding to 13.7% of the study population. The frequency distribution of individual clinical findings is presented in Table 5. Triceps abnormalities were the most frequently recorded finding, accounting for 35 cases (42.7% of all clinical findings), followed by lumbar abnormalities and diarrhoea, with 11 cases each (13.4%).

To facilitate interpretation of the clinical findings, individual abnormalities were subsequently grouped into three categories: traumatic injuries (including wounds, harness-related lesions, and paw-pad abrasions), musculoskeletal disorders (including lumbar, triceps, psoas, and shoulder abnormalities), and metabolic/systemic conditions (including fatigue, diarrhoea, and cardiac abnormalities).

Of the 82 recorded findings, 52 (63.4%) were classified as musculoskeletal disorders, 19 (23.2%) as metabolic/systemic conditions, and 11 (13.4%) as traumatic injuries.

The distribution of clinical finding categories differed significantly according to reproductive status (Pearson’s χ^2^ = 7.564, df = 2, *p* = 0.023). Among neutered dogs, 22 of 26 findings (84.6%) were classified as musculoskeletal, whereas 2 (7.7%) were traumatic and 2 (7.7%) were metabolic/systemic. In contrast, among intact dogs, 30 of 56 findings (53.6%) were musculoskeletal, 9 (16.1%) were traumatic, and 17 (30.4%) were metabolic/systemic.

Comparison of observed and expected frequencies showed that musculoskeletal findings were more frequent than expected among neutered dogs (22 observed vs. 16.5 expected cases), whereas metabolic/systemic findings were less frequent than expected (2 observed vs. 6.0 expected cases). Conversely, intact dogs presented fewer musculoskeletal findings (30 observed vs. 35.5 expected cases) and more metabolic/systemic findings (17 observed vs. 13.0 expected cases) than expected under the assumption of independence. The distribution of clinical finding categories according to reproductive status is summarised in Table 6.

Because neutered dogs were older than intact dogs in the overall study population, an additional exploratory analysis was performed to assess the potential influence of age on the distribution of musculoskeletal findings. When dogs presenting musculoskeletal abnormalities were analysed according to age group and reproductive status, no significant association was identified (Pearson’s χ^2^ = 1.243, df = 2, *p* = 0.537).

Overall, the distribution of clinical finding categories differed according to reproductive status, with musculoskeletal abnormalities representing a greater proportion of findings among neutered dogs and metabolic/systemic abnormalities representing a greater proportion of findings among intact dogs.

### 3.6. Factors Associated with Neutering Status

To identify factors associated with neutering status, a multivariable logistic regression model was fitted using championship, competition category (NB and OP), sex, breed, age group, and sled position variables. The model was statistically significant overall (χ^2^ = 73.82, df = 12, *p* < 0.001).

Sex was the strongest independent predictor of neutering status. Females were significantly more likely to be neutered than males (OR = 3.28, *p* < 0.001). Age was also associated with neutering status, with older dogs presenting significantly higher neutering rates than younger animals (overall *p* = 0.003). Competition category remained independently associated with neutering, with dogs competing in the OP category being less frequently neutered than those in the NB category (OR = 0.22, *p* = 0.002).

Breed showed a significant overall association with neutering status (*p* = 0.011). European Sled Dogs exhibited significantly different neutering patterns compared with Siberian Huskies. Neither championship nor sled position reached conventional statistical significance, although lead position showed a borderline association (*p* = 0.055).

The final regression model is presented in Table 7.

## 4. Discussion

This study provides one of the first field-based evaluations of neutering practices, reproductive management decisions, and health outcomes in actively competing European endurance sled dogs. By integrating demographic characteristics, musher-reported motivations, and veterinary findings obtained under real racing conditions, the study contributes novel information regarding reproductive management in canine endurance athletes. The principal findings were that neutering was relatively uncommon and was associated with female sex, older age, and breed category; that reproductive decisions reflected a combination of breeding, management, welfare, and performance-related considerations; and that, although neutering was not associated with an increased overall occurrence of clinical abnormalities, the distribution of clinical finding categories differed according to reproductive status. Specifically, intact dogs presented proportionally more metabolic abnormalities, whereas neutered dogs presented proportionally more musculoskeletal findings.

Long-distance sled dogs represent a unique population of highly conditioned canine athletes exposed to prolonged exercise, cumulative training loads, environmental stressors, and demanding management conditions [1,2,3,4]. Their physiological adaptations include substantial modifications in energy metabolism, exercise tolerance, and recovery mechanisms. Metabolomic studies have demonstrated marked shifts in substrate utilization during endurance racing, including increased lipid oxidation, amino acid depletion, and continued carbohydrate utilization, reflecting the remarkable metabolic flexibility required for prolonged athletic performance [8]. These characteristics make sled dogs particularly relevant for investigating factors that may influence both long-term health and athletic function.

### 4.1. Neutering Patterns in Actively Racing Sled Dogs

The prevalence of neutering observed in this population (28%) was markedly lower than that reported in many companion dog populations, supporting the concept that reproductive management in working and sporting dogs differs substantially from that of non-athletic companion animals. In competitive kennels, dogs frequently represent both athletic and genetic resources, and decisions regarding gonadectomy appear to be influenced by the need to preserve future breeding opportunities together with considerations related to performance, health, and kennel management.

Older dogs were significantly more likely to be neutered than younger individuals, suggesting that gonadectomy is often postponed until athletic potential and breeding value have been adequately assessed. Although age at surgery was unavailable, the observed pattern is consistent with management strategies in which reproductive decisions are delayed until dogs complete an important portion of their sporting careers. Similar associations between breeding intentions and neutering decisions have been documented in companion and working dog populations, in which perceived breeding value significantly reduces the likelihood of gonadectomy [11].

Female dogs were also more frequently neutered than males. This finding likely reflects health-related considerations such as prevention of pyometra and other reproductive disorders, together with the practical difficulties associated with managing oestrus in large mixed-sex racing teams. In contrast, intact males may be preferentially retained because of their potential breeding value and concerns regarding the possible influence of gonadal hormones on working capacity, trainability, endurance, and behaviour [12,21].

### 4.2. Reproductive Management Decisions in Racing Kennels

The motivations reported by mushers highlight the multifactorial nature of reproductive decision-making in canine athletes. Neutering was primarily justified by reproductive management, disease prevention, and behavioural considerations, reflecting the logistical challenges associated with maintaining multiple intact dogs under intensive training and competition schedules. Similar motivations have been reported in broader canine populations, where prevention of unwanted reproduction and anticipated health benefits are among the most common reasons for gonadectomy [11].

Conversely, decisions to maintain dogs intact were predominantly linked to breeding objectives, preservation of desirable genetic traits, perceived performance benefits, and ethical considerations. The prominence of breeding-related motivations is unsurprising given the importance of maintaining genetic lines associated with endurance, trainability, and working aptitude within successful racing kennels [22].

Interestingly, ethical concerns emerged as an independent reason for avoiding elective gonadectomy. This finding reflects an increasingly debated issue within veterinary medicine. While neutering has traditionally been promoted for population control and prevention of selected reproductive disorders [9,10], growing attention has been directed toward its potential long-term consequences on orthopaedic, metabolic, behavioural, endocrine, and neoplastic health outcomes [13,16,24].

Recent World Small Animal Veterinary Association (WSAVA) guidelines emphasize that reproductive management decisions should be individualized according to breed, age, lifestyle, intended use, and expected health outcomes rather than based on universal recommendations [24]. The present findings suggest that such individualized approaches may be particularly relevant in canine athletes, where long-term health, athletic longevity, breeding objectives, and performance expectations frequently coexist.

### 4.3. Clinical Findings During Competition

Approximately 14% of dogs presented at least one recorded clinical abnormality during competition, and most findings involved the musculoskeletal system. This pattern is consistent with previous studies identifying locomotor disorders as the principal health challenge in endurance sled dogs, both during competition and as a cause of race withdrawal [5,7,25].

Long-distance sled dog racing is associated with substantial physiological challenges affecting several organ systems [4]. Previous investigations of severe health events and race-related mortality have identified aspiration pneumonia, gastrointestinal disorders, gastric ulceration, and exercise-associated muscle diseases among the most important causes of morbidity and mortality in these dogs [5]. Orthopaedic injuries have also been recognised as a major welfare and performance concern, accounting for more than half of race withdrawals in some endurance competitions [7].

Importantly, the abnormalities recorded in the present study were predominantly mild and detected through routine veterinary surveillance rather than advanced diagnostic investigations. Trigger points, localised muscular discomfort, and minor soft-tissue abnormalities represented most observations. These findings likely reflect the cumulative biomechanical demands imposed by prolonged endurance exercise and repetitive locomotor loading. Because no imaging, laboratory testing, or biomechanical assessment was performed, their clinical significance and relationship with future injury remain uncertain.

Digestive and cardiologic abnormalities were uncommon and generally mild. Nevertheless, the low number of clinically apparent gastrointestinal findings should not be interpreted as evidence of limited gastrointestinal involvement during endurance racing. Previous endoscopic investigations conducted in racing sled dogs demonstrated a high prevalence of gastric ulceration, erosion, and gastric haemorrhage, even among dogs considered clinically healthy [6]. These findings indicate that routine clinical examinations may underestimate the true burden of exercise-associated gastrointestinal pathology and support the continued importance of systematic veterinary surveillance during competition.

More broadly, the present findings reinforce the distinction between clinical normality and physiological homeostasis in endurance athletes. Metabolomic studies have shown profound exercise-induced alterations in energy metabolism despite the absence of overt clinical disease, indicating that substantial physiological adaptation occurs during racing [8].

### 4.4. Neutering Status and Distribution of Clinical Abnormalities

Although neutering was not associated with an increased overall probability of presenting a clinical abnormality during competition, significant differences were identified in the distribution of clinical finding categories according to reproductive status. The most notable result was the differential distribution of metabolic and musculoskeletal abnormalities between neutered and intact dogs. Intact dogs exhibited a greater number of metabolic abnormalities than expected, whereas neutered dogs presented fewer metabolic findings than expected. Conversely, musculoskeletal disorders represented a greater proportion of the findings recorded in neutered dogs.

The higher frequency of metabolic abnormalities among intact dogs is particularly noteworthy in the context of endurance racing. The metabolic category included findings such as fatigue, gastrointestinal disturbances, and cardiac abnormalities, all of which may reflect the physiological demands imposed by prolonged exercise. One possible explanation is that intact dogs were exposed to greater physical demands during competition. In many racing kennels, intact dogs are retained because of their athletic and breeding value, and some mushers perceive them as possessing greater endurance capacity, drive, or competitive potential. If intact dogs were preferentially selected for more demanding work during racing, this could partially explain the greater occurrence of metabolic abnormalities observed in this group. However, objective measures of workload, exercise intensity, physiological strain, recovery status, and performance were unavailable in the present study, and this interpretation should therefore be considered speculative.

The predominance of musculoskeletal abnormalities among neutered dogs is broadly consistent with an expanding body of literature linking gonadectomy with altered musculoskeletal health. Several studies have reported increased risks of orthopaedic disorders, including cranial cruciate ligament disease, hip dysplasia, and elbow dysplasia, particularly when gonadectomy is performed before skeletal maturity [14,15,16]. More recently, a systematic review and meta-analysis involving more than 1.8 million dogs concluded that gonadectomy is associated with significantly increased odds of cranial cruciate ligament disease in both sexes [20]. The abnormalities recorded in the present study were considerably milder than those described in these investigations and consisted primarily of muscular dysfunction, localised discomfort, trigger points, and soft-tissue abnormalities. Nevertheless, the biological plausibility of the observed association is supported by the recognised influence of gonadal hormones on skeletal maturation, connective tissue physiology, muscle development, and musculoskeletal adaptation to exercise [13,24].

Alternative explanations must also be considered. Because neutered dogs were significantly older than intact dogs in the overall study population, age represented a potential confounding factor that could partially explain the greater proportion of musculoskeletal findings observed among neutered animals. To address this possibility, an additional exploratory analysis was performed among dogs presenting musculoskeletal abnormalities. No significant association was identified between age group and neutering status within this subset, and the observed frequencies closely matched those expected under the assumption of independence. These findings suggest that age alone is unlikely to explain the observed distribution of musculoskeletal abnormalities. Nevertheless, information regarding age at neutering, previous orthopaedic disease, body condition score, workload, and training exposure was unavailable. This limitation remains important because gonadectomy has been associated with an increased risk of overweight and obesity in several canine populations, and increased body weight may adversely affect musculoskeletal health through greater biomechanical loading and joint stress [19].

Consequently, the present results should not be interpreted as evidence of causality but rather as evidence of differing patterns of clinical abnormalities according to reproductive status in actively competing sled dogs. Further prospective studies incorporating objective performance metrics, training load assessment, body composition analysis, and age at gonadectomy are required to clarify the mechanisms underlying these associations and their relevance to the long-term health and athletic performance of canine endurance athletes.

### 4.5. Strengths and Limitations

A major strength of this study is the inclusion of a large population of actively competing sled dogs, a group that remains underrepresented in the scientific literature. The integration of reproductive management information with veterinary findings collected under real competition conditions provided a unique opportunity to evaluate neutering practices and health outcomes within a genuine athletic context.

Several limitations should nevertheless be acknowledged. First, the observational and cross-sectional design precludes causal inference. Consequently, the associations identified between reproductive status and the distribution of clinical findings should not be interpreted as evidence of a causal relationship.

Second, motivation was recorded as a single primary reason and therefore may not fully capture the complexity of reproductive decision-making. In addition, behavioural characteristics potentially relevant to neutering decisions, such as arousal, trainability, social interactions, or dominance-related behaviours, were not recorded.

The relatively small number of dogs presenting clinical abnormalities limited statistical power, particularly for analyses exploring associations between reproductive status and specific categories of clinical findings. Furthermore, information regarding previous medical history, prior injuries, age at neutering, training load, nutritional management, biomechanics, environmental exposure, body condition score, and body composition was unavailable. As a result, potentially important confounding factors influencing both reproductive status and health outcomes could not be evaluated.

Finally, all dogs were considered fit for competition at enrolment, introducing a potential healthy-athlete bias that may underestimate the true prevalence of health disorders within the broader sled dog population. In addition, multiple dogs originated from the same musher or kennel, potentially introducing clustering effects that could not be fully accounted for in the analyses.

### 4.6. Clinical Implications and Future Directions

For veterinarians working with sled dogs and other canine athletes, the present findings support an individualised approach to reproductive management. Decisions regarding gonadectomy should integrate health considerations, breeding objectives, athletic goals, long-term welfare, behavioural characteristics, and owner preferences rather than relying exclusively on evidence derived from companion animal populations.

From a sports medicine perspective, the results suggest that reproductive status may be associated with differing patterns of clinical abnormalities observed during endurance racing. Intact dogs presented proportionally more metabolic abnormalities, whereas neutered dogs presented proportionally more musculoskeletal findings. Although the mechanisms underlying these differences remain unclear, these observations highlight the importance of comprehensive veterinary monitoring that encompasses both locomotor and metabolic health during competition.

The predominance of mild musculoskeletal abnormalities reinforces the importance of workload management, conditioning programmes, injury surveillance, and routine veterinary examinations as key components of preventive healthcare in canine endurance athletes. Equally, the higher-than-expected frequency of metabolic abnormalities among intact dogs suggests that particular attention should be paid to monitoring fatigue, gastrointestinal disturbances, hydration status, and other indicators of physiological strain during competition.

Future prospective studies incorporating objective measures of training load, exercise intensity, performance, recovery status, gait analysis, body composition, and musculoskeletal function are needed to clarify the determinants of health outcomes in endurance sled dogs. Longitudinal investigations following dogs throughout multiple racing seasons would be particularly valuable for determining whether reproductive status, age at gonadectomy, body composition, or cumulative racing exposure influence athletic longevity, injury risk, metabolic resilience, or long-term musculoskeletal health.

Such studies are necessary to establish a stronger evidence base for reproductive management decisions in working and sporting dogs and to improve our understanding of the interaction among reproductive status, metabolic adaptation, musculoskeletal function, and athletic performance in canine endurance athletes.

## 5. Conclusions

This study provides a field-based characterisation of neutering practices, reproductive management decisions, and clinical findings in a large population of actively competing European sled dogs. Neutering was relatively uncommon, affecting 28% of the study population, and was more frequently observed in females and older dogs, reflecting the combined influence of breeding objectives, reproductive management, health considerations, and kennel management practices.

Most clinical abnormalities recorded during competition were mild and involved the musculoskeletal system, confirming the predominance of locomotor disorders in canine endurance athletes. Reproductive status was not associated with the overall occurrence of clinical abnormalities during competition, suggesting that neutering status alone is unlikely to be a major determinant of short-term clinical outcomes in actively racing sled dogs.

However, reproductive status was associated with the distribution of clinical finding categories. Intact dogs presented proportionally more metabolic abnormalities, whereas neutered dogs presented proportionally more musculoskeletal findings. These results indicate that the pattern of clinical abnormalities observed during endurance racing may differ according to reproductive status, although the mechanisms underlying these associations remain unclear.

Given the observational nature of the study, no causal relationship can be inferred. Nevertheless, the findings highlight the need for further prospective investigations incorporating age at gonadectomy, training load, body composition, performance metrics, previous injury history, and objective measures of physiological and biomechanical responses to exercise. Such studies are required to clarify whether reproductive status is associated with long-term differences in metabolic adaptation, musculoskeletal health, athletic longevity, or performance in canine endurance athletes.

Overall, the present results support an individualised approach to reproductive management in athletic dogs, consistent with current WSAVA recommendations, whereby decisions regarding gonadectomy should consider breed, age, intended use, athletic demands, long-term health considerations, and owner objectives rather than relying on universal recommendations.

## Figures and Tables

**Table 1:** Demographic and competition characteristics of sled dogs participating in the 2023 Lekkarod^®^ event (*n* = 599).

Variable	Category	Frequency (%)
Sex	Female	274 (45.7)
	Male	325 (54.3)
Competition category	NB	319 (53.3)
	OP	280 (46.7)
Breed	Siberian Husky (SH)	312 (52.1)
	Alaskan Husky (AH)	166 (27.7)
	European Sled Dog (ESD)	100 (16.7)
	Other breeds	21 (3.5)
Reproductive status	Neutered	168 (28.0)
	Intact	431 (72.0)
Age (days)	Mean ± SD	1782 ± 841
	Range	555–4284
Age (years)	Mean ± SD	4.9 ± 2.3
	Range	1.5–11.7

Abbreviations: NB, Nordic Breed category; OP, Open category; SH, Siberian Husky; AH, Alaskan Husky; ESD, European Sled Dog; SD, standard deviation.

**Table 2:** Distribution of team positions among sled dogs with available positional information (*n* = 550).

Team Position	Frequency (%)
Lead	162 (29.5)
Wheel	192 (34.9)
Swing/Team	196 (35.6)
Total	550 (100)

Note: Positional information was unavailable for 49 dogs (8.2%).

**Table 3:** Primary motivations reported for maintaining dogs intact.

Motivation Category	Frequency (%)
Breeding and genetic preservation	179 (41.5)
No intervention considered necessary	114 (26.5)
Performance-related considerations	42 (9.7)
No perceived benefit of neutering	38 (8.8)
Ethical concerns	2 (0.5)
Other reasons	56 (13.0)
Total	431 (100.0)

Note: Percentages were calculated using the number of intact dogs for which motivation data were available.

**Table 4:** Primary motivations reported for neutering.

Motivation Category	Frequency (%)
Oestrus and reproductive management	81 (48.2)
Behavioural management	32 (19.0)
Disease prevention	25 (14.9)
Group-dynamics management	25 (14.9)
Medical indication	11 (6.5)
Other reasons	24 (14.3)

Note: Percentages were calculated using the number of neutered dogs for which motivation data were available.

**Table 5:** Distribution of individual clinical findings recorded during the 2023 Lekkarod^®^ event (*n* = 82 clinical findings).

Clinical Finding	Frequency	%	Cumulative %
Wounds	5	6.1	6.1
Fatigue	5	6.1	12.2
Diarrhoea	11	13.4	25.6
Paw-pad abrasions	6	7.3	32.9
Cardiac abnormalities	3	3.7	36.6
Lumbar abnormalities	11	13.4	50.0
Triceps abnormalities	35	42.7	92.7
Psoas abnormalities	5	6.1	98.8
Shoulder abnormalities	1	1.2	100.0
Total	82	100.0	—

**Table 6:** Distribution of clinical finding categories according to reproductive status in sled dogs participating in the 2023 Lekkarod^®^ event (*n* = 82 clinical findings).

Reproductive Status	Traumatic n (%)	Musculoskeletal n (%)	Metabolic/Systemic n (%)	Total
Neutered (observed)	2 (7.7)	22 (84.6)	2 (7.7)	26
Neutered (expected)	3.5	16.5	6.0	26
Intact (observed)	9 (16.1)	30 (53.6)	17 (30.4)	56
Intact (expected)	7.5	35.5	13.0	56
Total	11 (13.4)	52 (63.4)	19 (23.2)	82

Note: Percentages represent the proportion of each clinical finding category within each reproductive status group. Expected values correspond to frequencies under the assumption of independence. The distribution of clinical finding categories differed significantly according to reproductive status (Pearson’s χ^2^ = 7.564, df = 2, *p* = 0.023).

**Table 7:** Multivariable logistic regression analysis of factors associated with neutering status.

Variable	Odds Ratio (OR)	*p* Value
Championship		
Kraft vs. Lekkarod	0.66	0.053
Competition category		
OP vs. NB	0.22	0.002
Sex		
Female vs. Male	3.28	<0.001
Breed (overall effect)	—	0.011
AH vs. SH	2.13	0.130
ESD vs. SH	5.76	0.001
Team position		
Lead position (Yes vs. No)	1.63	0.055
Wheel position (Yes vs. No)	1.03	0.913
Age group (overall effect)	—	0.003
Middle-aged vs. Young	0.58	0.037
Old vs. Young	0.41	<0.001

Abbreviations: OR, odds ratio; SH, Siberian Husky; AH, Alaskan Husky; ESD, European Sled Dog. Exact 95% confidence intervals could not be reported because they were not generated in the original SPSS output.

## Data Availability

The data supporting the findings of this study are available from the corresponding author upon reasonable request. The data are not publicly available due to privacy and confidentiality considerations related to participating dog owners, handlers, and kennels.

## Abbreviations

The following abbreviations are used in this manuscript:

AH	Alaskan Husky
ESD	European Sled Dog
NB	Nordic Breed category
OP	Open category
OR	Odds Ratio
SD	Standard Deviation
SH	Siberian Husky
WSAVA	World Small Animal Veterinary Association
